# KDM1A/LSD1 regulates the differentiation and maintenance of spermatogonia in mice

**DOI:** 10.1371/journal.pone.0177473

**Published:** 2017-05-12

**Authors:** Dexter A. Myrick, Michael A. Christopher, Alyssa M. Scott, Ashley K. Simon, Paul G. Donlin-Asp, William G. Kelly, David J. Katz

**Affiliations:** 1 Cell Biology Department, Emory University, Atlanta, Georgia, United States of America; 2 Graduate Division of Biological and Biomedical Science, Emory University, Atlanta, Georgia, United States of America; 3 Biology Department, Emory University, Atlanta, Georgia, United States of America; University Hospital of Münster, GERMANY

## Abstract

The proper regulation of spermatogenesis is crucial to ensure the continued production of sperm and fertility. Here, we investigated the function of the H3K4me2 demethylase KDM1A/LSD1 during spermatogenesis in developing and adult mice. Conditional deletion of *Kdm1a* in the testis just prior to birth leads to fewer spermatogonia and germ cell loss before 3 weeks of age. These results demonstrate that KDM1A is required for spermatogonial differentiation, as well as germ cell survival, in the developing testis. In addition, inducible deletion of *Kdm1a* in the adult testis results in the abnormal accumulation of meiotic spermatocytes, as well as apoptosis and progressive germ cell loss. These results demonstrate that KDM1A is also required during adult spermatogenesis. Furthermore, without KDM1A, the stem cell factor OCT4 is ectopically maintained in differentiating germ cells. This requirement for KDM1A is similar to what has been observed in other stem cell populations, suggesting a common function. Taken together, we propose that KDM1A is a key regulator of spermatogenesis and germ cell maintenance in the mouse.

## Introduction

In mammals, sperm are continuously produced over the lifetime of adult males. This continuous production of sperm is maintained by the ongoing differentiation of spermatogonia [1]. Recently, the histone demethylase KDM1A (lysine specific demethylase 1A) has been implicated in the differentiation of multiple cell types [2–4]. Therefore, to gain insight into the mechanism of spermatogonial differentiation, we investigated the function of KDM1A in mouse spermatogenesis.

In male mice, primordial germ cells colonize the developing testis and become prospermatogonia or gonocytes (hereafter referred to as prospermatogonia) by embryonic day 12.5 (E12.5). After birth, these prospermatogonia become undifferentiated spermatogonia and also transition directly to differentiated spermatogonia. The differentiated spermatogonia then become haploid spermatozoa and complete the first wave of spermatogenesis, which takes approximately 35 days [5]. Following the first wave of spermatogenesis, the undifferentiated spermatogonia continue to undergo meiosis and produce mature spermatozoa [1]. This process occurs continuously throughout the lifetime of adult males.

Accumulating evidence has implicated the histone modification di-methylation of lysine 4 on histone H3 (H3K4me2) in the maintenance of transcriptional states during development [6–9]. However, if H3K4me2 functions in the maintenance of transcription, then this histone methylation may have to be reprogrammed to allow for changes in cell fate. This function may be accomplished by the activity of histone demethylases such as KDM1A. KDM1A is an amine-oxidase type histone demethylase that is part of the CoREST (co RE1-silencing transcription factor) complex and specifically demethylates H3K4me2 *in vitro* [10, 11]. KDM1A associates with CoREST in pachytene spermatocytes [12], though it is unknown whether it also interacts with CoREST in other germ cells. KDM1A has also been shown to associate with the androgen receptor (AR) complex in the mouse testis [13]. When associated with the AR complex *in vitro*, KDM1A has specificity for H3K9me2 [13].

In mammals, loss of KDM1A in the mouse embryo results in embryonic lethality prior to E7, when tissues are first beginning to be specified [14, 15]. Furthermore, KDM1A has been implicated in the differentiation of several mouse cell types *in vitro* [16–19] and in the terminal differentiation of pituitary cells during pituitary organogenesis *in vivo* [14]. Recently, KDM1A has also been demonstrated to have a role in stem cell differentiation [2–4]. During the differentiation of mouse ES cells *in vitro*, KDM1A is required to remove H3K4 methylation at the promoters and enhancers of stem cell genes [3]. For example, KDM1A binds to the promoter, as well as the proximal and distal enhancers, of the critical stem cell gene *Oct4*. When KDM1A is depleted, H3K4 methylation at these stem cell genes is not properly removed and the expression of these genes is inappropriately maintained during mES cell differentiation [3]. This may cause the differentiation defect observed in KDM1A-depleted mouse ES cells [3, 15, 20]. In addition, KDM1A is thought to act in a similar fashion during hematopoietic stem cell differentiation *in vivo* in the mouse [2].

In the testis, *Oct4* is expressed in undifferentiated spermatogonia. It is required for the maintenance of spermatogonia *in vitro* and facilitates the colonization of the testis following spermatogonial transplantation *in vivo* [21]. In addition, KDM1A has been shown to directly bind *Oct4* in a mouse germ cell line (GC-1 cells)[22]. Since KDM1A has been implicated in the transcriptional repression of critical transcription factors, such as *Oct4*, during stem cell differentiation, we hypothesized that KDM1A may also be required for differentiation during spermatogenesis. Consistent with this hypothesis, Lambrot et al. recently provided the first evidence that KDM1A functions during the first wave of spermatogenesis in the maintenance and differentiation of spermatogonia [23]. Using the identical *Kdm1a* conditional deletion mouse, our findings agree with these conclusions. In addition, we extended these findings by utilizing a tamoxifen inducible *Cre* allele to analyze the function of KDM1A in adult spermatogenesis. This analysis suggests that KDM1A has an ongoing role in adult spermatogenesis. Finally, our data suggest that KDM1A regulates the transcription of *Oct4* during spermatogonial differentiation. Taken together, our results provide further evidence that KDM1A is a key regulator of spermatogenesis in mice.

## Results

### KDM1A is dynamically expressed in the murine testis

**Fig 1:**
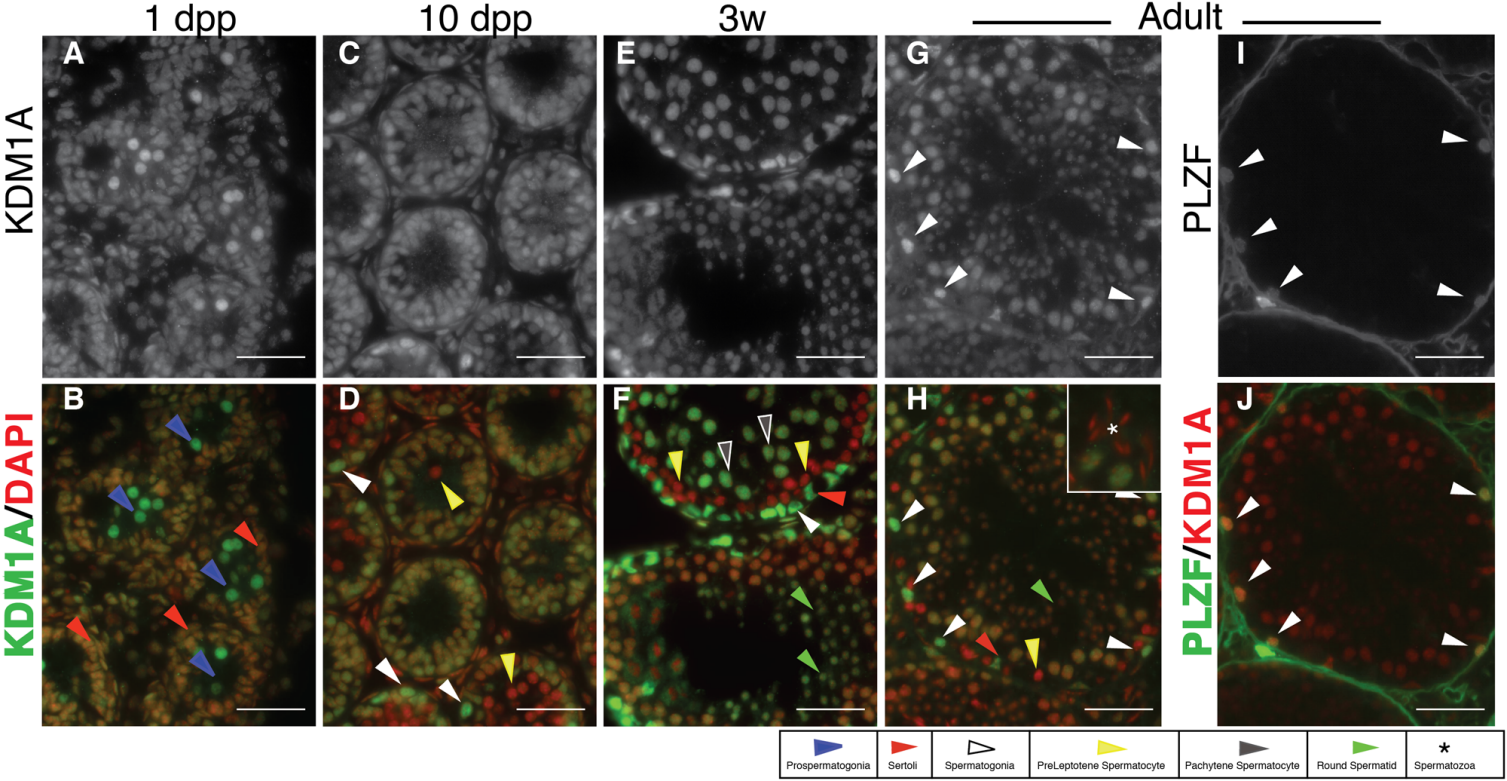
Expression of KDM1A in the testis. KDM1A (A,C,E,G), PLZF (I) and combined DAPI (red) and KDM1A (green) (B,D,F,H) immunofluorescence from wild-type testes. (J) Combined PLZF (green) and KDM1A (red) IF. Asterisk in magnified inset (H) indicates mature spermatozoa. White arrowheads in (I,J) indicate spermatogonia marked by PLZF. The expression of KDM1A in these same PLZF+ spermatogonia is shown in G,H (white arrowheads). In all other panels, spermatogenic cell types are labeled as described in legend (dpp = days post partum). Cell types were identified based on morphology and location within the testicular cord or seminiferous tubule. Scale bars, 25 μm.

We first asked if KDM1A protein is present in prospermatogonia and spermatogonia. Consistent with prior observations [23, 24], immunofluorescence with a KDM1A antibody demonstrates that KDM1A protein is found in prospermatogonia and Sertoli Cells in testicular cords at 1 day post partum (dpp) (Fig 1A and 1B). In addition, in juvenile testes KDM1A is present in spermatogonia, as well as in Sertoli cells (Fig 1C and 1D). KDM1A is subsequently absent in preleptotene spermatocytes, and then present again in pachytene spermatocytes and round spermatids, but not in mature spermatozoa (Fig 1C–1F, inset Fig 1H). Also, consistent with previously reported immunohistochemistry [24], this localization pattern is the same in adult testes (Fig 1G–1J). In particular, in adult mice KDM1A is present in PLZF positive spermatogonia (Fig 1I and 1J). KDM1A is also present in adult Sertoli Cells (Fig 1G and 1H).

### Loss of KDM1A causes defects in the maintenance and differentiation of spermatogonia

**Fig 2:**
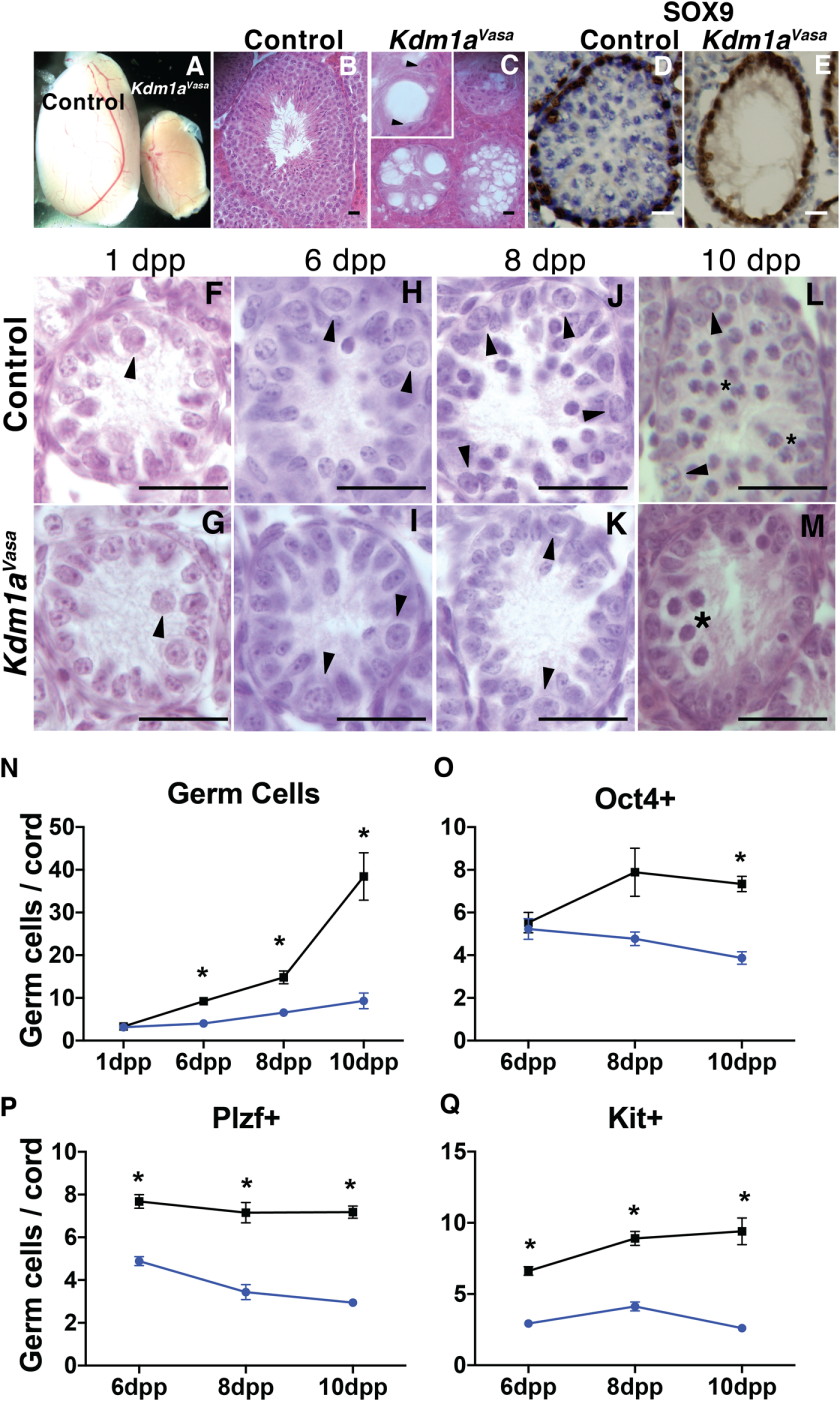
Spermatogonia differentiation and maintenance defect in *Kdm1a*^*Vasa*^ mutants. (A) Representative images of control and *Kdm1a*^*Vasa*^ mutant adult testes. Histology from control (B) and *Kdm1a*^*Vasa*^ (C) adult testes. Arrowheads indicate Sertoli cells (C). SOX9 immunohistochemistry (brown) counterstained with hematoxylin (blue) from control (D) and *Kdm1a*^*Vasa*^ (E) testes at 21days post partum (dpp). Histology from control (F,H,J,L) and *Kdm1a*^*Vasa*^ testes (G,I,K,M) at 1dpp (F,G), 6dpp (H,I), 8dpp (J,K) and 10dpp (L,M). Arrowheads indicate spermatogonia (F-L), and asterisk indicates spermatocytes (L) and abnormal spermatocytes (M). (N) Germ cells per testis cord quantified from histology (F,G) at 1dpp, and SOX9 immunofluorescence (S2 Fig) at 6dpp, 8dpp and 10dpp (the adult testis size was not quantified). Quantification of OCT4+ (O), PLZF+ (P) and KIT+ (Q) germ cells per cord from immunofluorescence (S1 Fig) (n = >30 testis cords counted from multiple animals, Mann-Whitney U test, p < .001). (N-Q) The control is shown in black and Kdm1a^Vasa^ is shown in blue. Scale bars, 25 μm.

To determine the role of KDM1A in spermatogenesis we conditionally deleted *Kdm1a* by crossing floxed *Kdm1a* mice [14] to a *Ddx4*/*Vasa-Cre* transgenic line [25] and a tamoxifen inducible *Cagg-Cre* transgenic line [26]. The resulting *Kdm1a*^*flox/flox*^;*Vasa-Cre* and *Kdm1a*^*flox/flox*^;*Cagg-Cre* mice are hereafter referred to as *Kdm1a*^*Vasa*^ and *Kdm1a*^*Cagg*^. Littermate *Kdm1a*^*flox/+*^;*Vasa-Cre* or *Kdm1a*^*flox/flox*^ without *Vasa-Cre* are used as controls in all subsequent *Kdm1a*^*Vasa*^ experiments. Tamoxifen-injected *Cre* minus littermates are used as controls in all subsequent *Kdm1a*^*Cagg*^ experiments. In comparison to endogenous *Vasa*, which is expressed earlier, *Vasa-Cre* is strongly induced in the germline between E15 and E18, with near complete recombination occurring by birth [25]. In the male germline, prospermatogonia are fully established well before the onset of *Vasa-Cre* [27]. As a result, deletion of *KDM1A* with *Vasa-Cre* can be used to determine the role of KDM1A in the differentiation and maintenance of germ cells. *Kdm1a*^*Vasa*^ males are sterile and exhibit a dramatic reduction in the size of adult testes (Fig 2A). To further investigate this phenotype, we examined histology and markers in control and *Kdm1a*^*Vasa*^ testes at 1, 6, 8, 10 and 21dpp, as well as in adults (Fig 2A–2Q, S1A–S1R and S2A–S2H Figs). Without KDM1A, adult testes lack germ cells (Fig 2B and 2C), and immunohistochemistry with the Sertoli cell marker SOX9 confirms that germ cells are lost prior to 21dpp (Fig 2D and 2E). Thus, KDM1A is required to maintain germ cells, including spermatogonia.

Examination of *Kdm1a*^*Vasa*^ testes at earlier time points suggests that the loss of germ cells is due to a defect in both spermatogonial differentiation and maintenance. At 1dpp, *Kdm1a*^*Vasa*^ testicular cords contain the same number of germ cells as controls (Fig 2F, 2G and 2N). Thus, as expected from the timing of *Vasa-Cre* expression, KDM1A does not affect the specification of prospermatogonia. In control 6dpp testes, we observe OCT4+ undifferentiated spermatogonia and KIT+ differentiating spermatogonia. (Fig 2H, 2O, 2Q and S1A and S1M Fig). In mutants at 6dpp we observe identical numbers of OCT4+ undifferentiated spermatogonia (Fig 2I and 2O and S1B Fig). Thus, without KDM1A, OCT4+ spermatogonia are normal. However, by 6dpp, *Kdm1a*^*Vasa*^ mutants show the first signs of a defect in the maintenance of undifferentiated spermatogonia. This is indicated by the lower numbers of PLZF+ spermatogonia compared to controls (Fig 2P and S1G and S1H Fig). At 6dpp there is also a defect in spermatogonial differentiation, as indicated by the lower numbers of KIT+ differentiating spermatogonia, as well as an overall decrease in germ cell number (Fig 2H, 2I, 2N, 2Q and S1M, S1N, S2A and S2B Figs). At 8dpp and 10dpp, there continues to be fewer PLZF+ spermatogonia (Fig 2P and S1I–S1L Fig). Also, by 10dpp there is now a significant decrease in the number of OCT4+ spermatogonia compared to controls (Fig 2O and S1C–S1F Fig). This indicates a progressive defect in the maintenance of undifferentiated spermatogonia (Fig 2J–2M). Furthermore, at 8dpp we continue to observe far fewer KIT+ differentiating spermatogonia (Fig 2Q and S1O and S1P Fig). Finally, by 10dpp in many control testicular cords spermatogonia have differentiated to produce spermatocytes and there is a rapid expansion in the number of germ cells (Fig 2L and 2N and S2E Fig). In contrast, *Kdm1a*^*Vasa*^ mutant cords contain very few spermatocytes, and even at later stages, post-meiotic spermatids are never observed (Fig 2M and 2N and S2F Fig).

**Fig 3:**
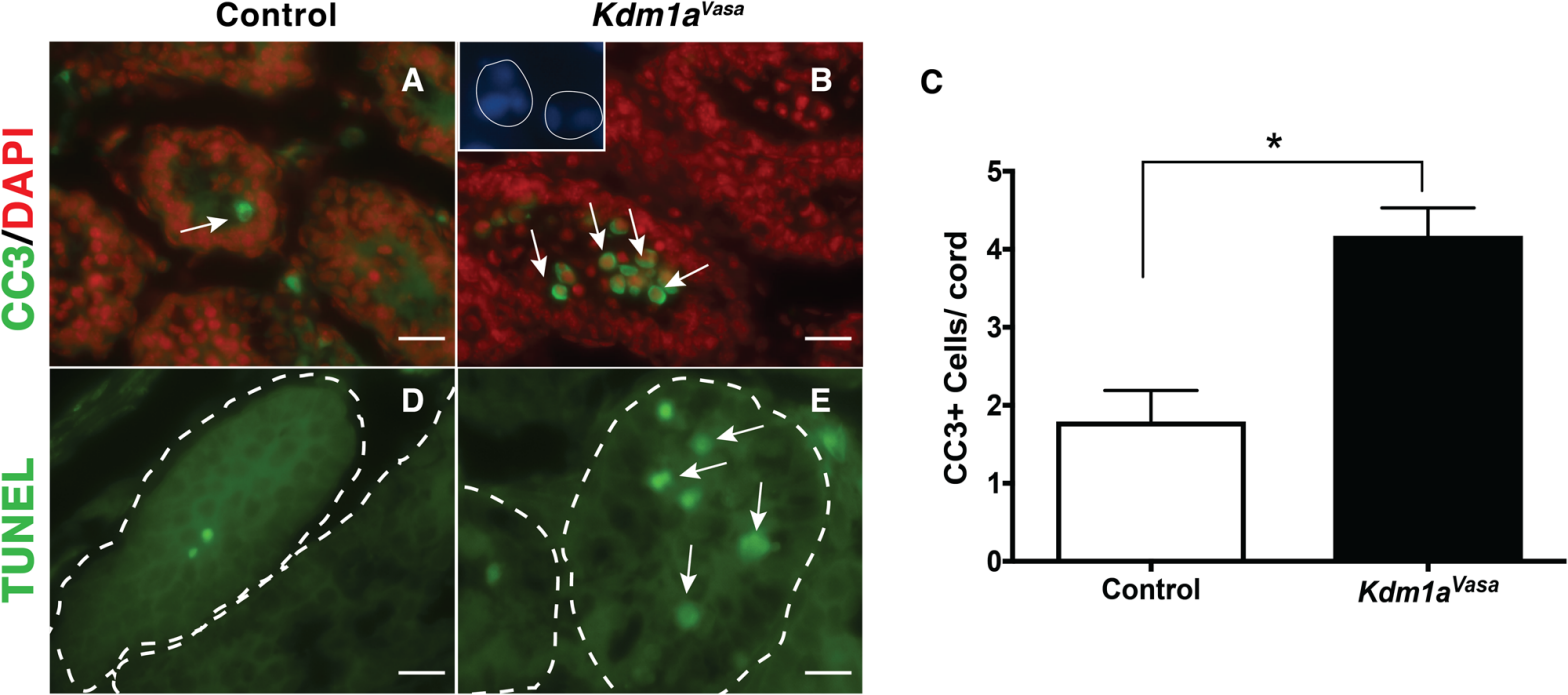
Germ cell apoptosis in *Kdm1a*^*Vasa*^ mutants. Quantification of Cleaved Caspase-3 (CC3) positive germ cells per testicular cord (C) from control (A) and *Kdm1a*^*Vasa*^ (B) testes (n = >30 testis cords counted from multiple animals), Mann-Whitney U test, p < .001). Arrows (A,B) indicate CC3 positive nuclei. Magnified inset (B) shows fragmented DNA in germ cells from a different Kdm1a^Vasa^ testis (DAPI: blue). TUNEL (green) from control (D) and *Kdm1a*^*Vasa*^ mutants (E) at 10dpp. Testicular cord boundaries are indicated by dashed lines. Scale bars, 25 μm.

At 10dpp, we also observe germ cell apoptosis. For example, over 40% (*Kdm1a*^*Vasa*^ average: 4.2 vs control average: 1.8 per cord) of the remaining germ cells in *Kdm1a*^*Vasa*^ testes are positive for the apoptosis marker Cleaved Caspase-3 (Fig 3A–3C) and many of these germ cells are TUNEL positive (Fig 3D and 3E). We also observe the apoptosis hallmark, fragmented DNA, in some remaining germ cells (inset Fig 3B). Overall, the combination of spermatogonia maintenance defect, differentiation defect and germ cell apoptosis results in a large deficit in the number of germ cells at 10dpp (Fig 2L–2N and S2E and S2F Fig) and the loss of germ cells by 21dpp (Fig 2D and 2E and S2G and S2H Fig).

*Kdm1a*^*Vasa*^ males lack germ cells by 21dpp and never complete a spermatogenic cycle. Therefore, to better understand the fate of germ cells in *Kdm1a*^*Vasa*^ mutants, we conditionally deleted *Kdm1a* with the tamoxifen inducible *Cagg-Cre* transgene in adult mice [26]. Since the *Cagg-Cre* transgene is active throughout the adult mouse somatic cells in the testis, as well as other tissues, may be affected. Nevertheless, since spermatogenesis is continuously ongoing in the adult male, the inducibility of the *Cre* transgene enabled us to determine if KDM1A has an ongoing function during spermatogenesis in adults.

**Fig 4:**
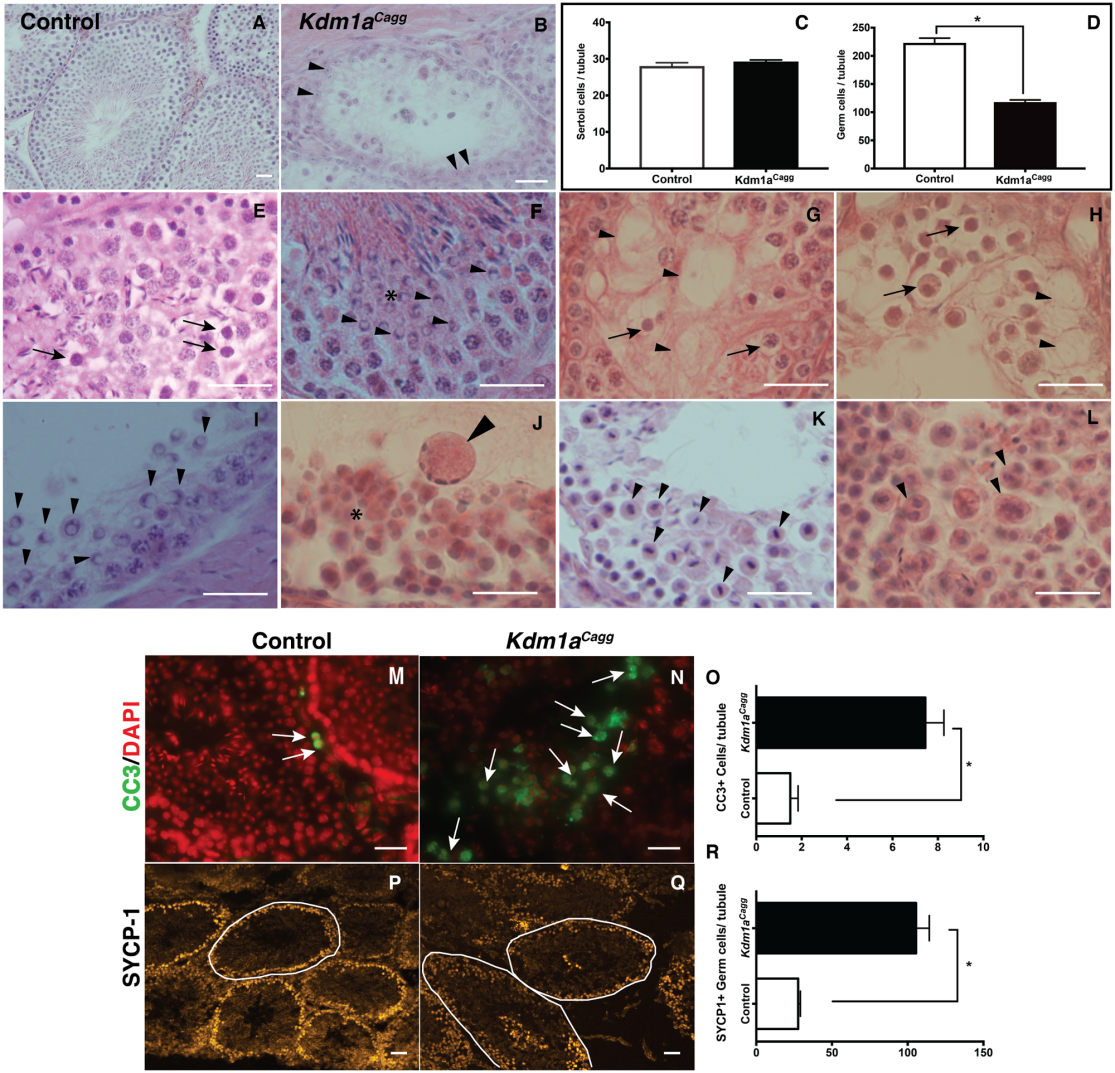
Germ cell maintenance and meiotic defects in *Kdm1a*^*Cagg*^ adult testes. Histology from control (A) and *Kdm1a*^*Cagg*^ testes (B,E-L), 7–9 weeks after the last tamoxifen injection to *delete Kdm1a*, showing mostly Sertoli cells remaining (arrowheads in B), spematogonia-like cells near or in the lumen (arrows in E), seminiferous tubule lacking a lumen (E,L), abnormally clumped germ cells (asterisk in F,J), abnormal spacing around cells (arrow in G,H) with vacuoles interspersed (arrowheads in G,H), germ cells with crescent shaped apoptotic morphology (arrowheads in F,I), giant spermatocyte-like apoptotic cells (arrowhead in J), chromosomal abnormalities (arrowheads in K), and multi-nucleated germ cells (arrowheads in L). Quantification of SOX9+ Sertoli cells (C) and germ cells per seminiferous tubule (D) in control and *Kdm1a*^*Cagg*^ testis (n = 3, controls and n = 4, *Kdm1a*^*Cagg*^ mutants, >25 seminiferous tubules per animal, Mann-Whitney U test, p < .05). Apoptosis marker CLEAVED CASPASE-3 (CC3)(green) merged with DAPI (red) (M,N) and meiotic marker SYCP-1 (yellow) (P,Q) and from control (M,P) and *Kdm1a*^*Cagg*^ mutants (N,Q). Quantification of the CC3 (O) and SYCP-1 (R) immunofluorescence (n = >30 seminiferous tubules counted from multiple animals, Mann-Whitney U test, p < .05). Scale bars, 25 μm.

Using a similar inducible deletion approach with another spermatogonia expressed gene, *Nanos2*, it has been demonstrated that 12 weeks is sufficient to cause a complete loss of all germ cells [28]. However, at time points beyond 9 weeks, *Kdm1a*^*Cagg*^ mice begin to have defects in the nervous system and die (M. Christopher, D. Myrick et al., reported elsewhere). As a result, analyses of *Kdm1a*^*Cagg*^ testes were done at or before 9 weeks to avoid complications due to these defects. At 9 weeks after the last tamoxifen injection the most affected tubules contain mostly Sertoli cells (Fig 4A and 4B). This demonstrates that KDM1A has an ongoing role in adult spermatogenesis. Also, the observation that Sertoli cells remain (Fig 4B and 4C) and are morphologically normal in even the most affected seminiferous tubules, indicates that these cells may not be affected, even though these cells express KDM1A (Figs 1 and 4B). However, future studies will be necessary to determine definitively whether this is the case. Approximately 7–9 weeks after the last tamoxifen injection, the majority of tubules are highly disorganized, with many germ cells abnormally clumped (Fig 4E, 4F and 4J) or out of place. In particular, we observe spematogonia-like cells near or in the lumen (Fig 4E), abnormal spacing around cells with vacuoles interspersed (Fig 4G and 4H), and some tubules lacking a lumen altogether (Fig 4E and 4L).

In *Kdm1a*^*Cagg*^ testes, we also observe defects that are similar to what we observe during the first wave of spermatogenesis. For example, many germ cells display the classic crescent shaped apoptotic morphology (Fig 4F and 4I). These crescent shaped nuclei are not observed in tamoxifen injected littermate controls. To confirm that these cells are undergoing apoptosis, we performed immunofluorescence with the apoptosis marker Cleaved Caspase-3 (Fig 4M and 4N). This analysis demonstrated a 4.5-fold increase in the number of apoptotic nuclei compared to controls (Fig 4O). We also observe many tubules with far fewer germ cells than normal (Fig 4B and 4G–4I). Quantification of germ cells demonstrated a 1.8-fold (*Kdm1a*^*Cagg*^: 120/tubule vs. control: 221/tubule) decrease in germ cells (Fig 4D). There are also many giant spermatocyte-like apoptotic cells present (Fig 4J). Furthermore, in *Kdm1a*^*Cagg*^ testes we observe many germ cells with chromosomal abnormalities and multi-nucleated germ cells (Fig 4K and 4L). These defects indicate a potential block to meiotic entry. To confirm this block, we performed immunofluorescence with the meiotic marker synaptonemal complex protein 1 (SYCP-1)(Fig 4P and 4Q). This analysis demonstrated a 3.8-fold (*Kdm1a*^*Cagg*^:105.5/tubule vs. control: 27.7/tubule) increase in the number of SYCP-1 positive nuclei compared to tamoxifen-injected littermate controls, indicating arrest during early meiotic prophase (Fig 4R).

### Loss of KDM1A does not derepress *Line1* and *IAP* retrotransposons

*Miwi2* and *Dnmt3l* mutant mice exhibit a loss of germ cells that is similar to *Kdm1a* mutants [29, 30]. MIWI2 is an RNA binding protein of the *Piwi* family that functions in the production of germline PIWI interacting RNA’s (piRNAs) [29]. Without MIWI2, germ cells arrest in meiosis and seminiferous tubules degenerate over time. This ultimately results in the loss of germ cells in adult males [29]. DNMT3L associates with the *de novo* methyltransferase complex and is required for proper *de novo* methylation in mammals [31]. Without *Dnmt3l*, male mice exhibit severe defects that are identical to *Miwi2* [30]. The testis phenotypes in both *Miwi2* and *Dnmt3l* mutants are thought to be caused by the reactivation of retrotransposons, leading to meiotic catastrophe. Specifically, in *Dnmt3l* and *Miwi2* mutants, *Line1* and *IAP* retrotransposons fail to properly acquire DNA methylation and are inappropriately expressed [29, 30]. Therefore, to determine if the *Kdm1a* mutant phenotype could be due to a similar mechanism, we examined DNA methylation and the expression of *Line1* and *IAP* elements in *Kdm1a*^*Vasa*^ and *Kdm1a*^*Cagg*^ testes. In *Kdm1a*^*Cagg*^ testes, we do not detect any expression of *IAP* retrotransposons (S3A and S3B Fig). Nor do we detect a decrease in DNA methylation at *Line1* or *IAP* elements (S3E Fig). We do observe *Line1* expression in spermatocytes of *Kdm1a*^*Cagg*^ testes (S3C Fig). However, we also detect this same expression in control adult testes (S3D Fig). To our knowledge, this is the first time that the expression of *Line1* retrotransposons has been examined in spermatocytes of adult seminiferous tubules [32, 33]. This expression is surprising since retrotransposition in these gametes could have a large negative impact. We also do not observe increased expression of *Line1* elements, or any decrease in DNA methylation at *Line1* and *IAP* elements in *Kdm1a*^*Vasa*^ mutants at 10dpp (S3F–S3H Fig). Importantly, because the methylation analysis was performed on whole testis in one control versus one mutant, it is possible that loss of *IAP* or *Line1* methylation could have been missed in a specific testis cell type. Nevertheless, the unaffected methylation pattern is consistent with the lack of expression defect, which was performed in a cell type specific fashion. Based on the similarity of the germ cell loss and meiotic progression defect with those observed in *Miwi2* and *Dnmt3l* mutants, it was possible that the *Kdm1a* functions as part of the mechanism to regulate transposons in the testis. However, the overall lack of retrotransposon defects indicates that this is not likely.

### KDM1A binds to the *Oct4* locus in the adult testis

**Fig 5:**
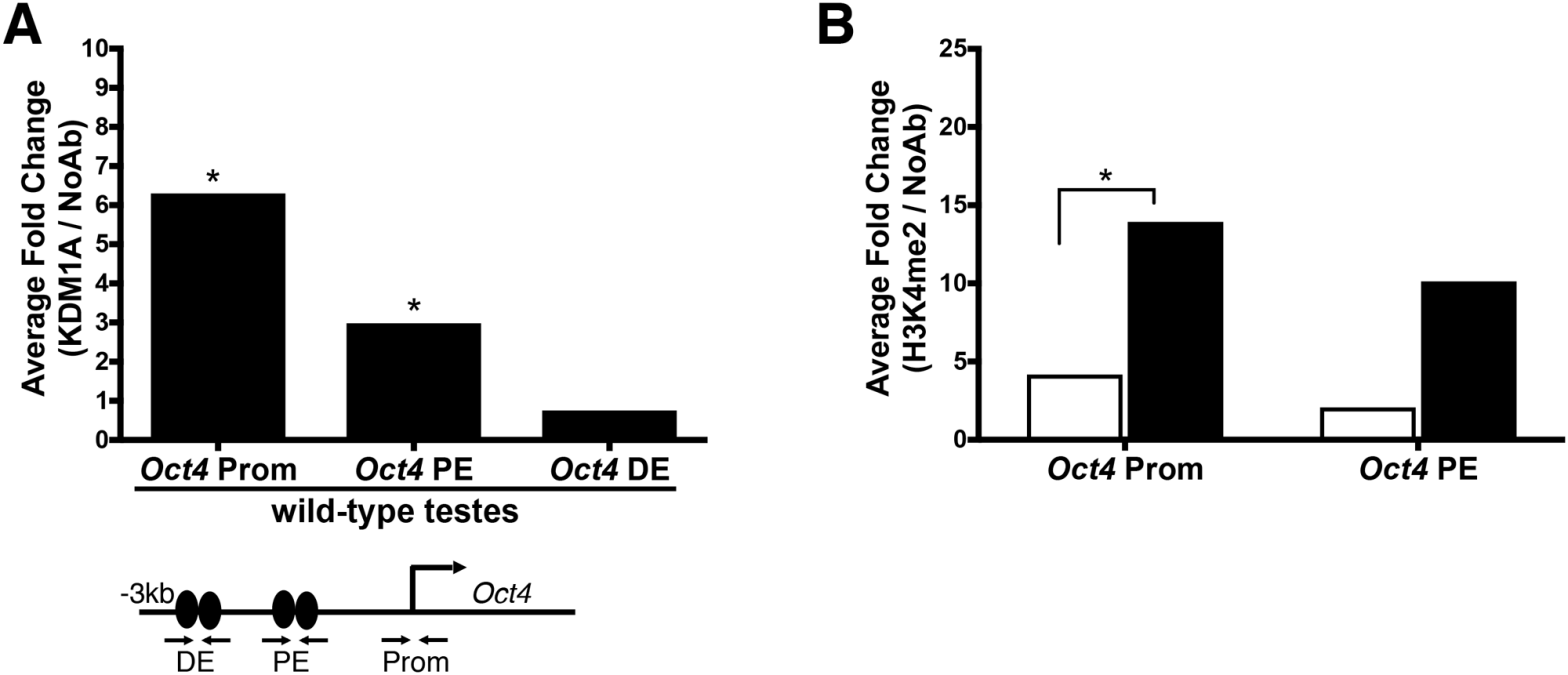
KDM1A and H3K4me2 chromatin immunoprecipitation at *Oct4*. (A) Average fold enrichment (KDM1A/no Ab) from chromatin immunoprecipitation (ChIP) at *Oct4* in wild-type adult testes calculated from S4A Fig (n = 2, Unpaired t-test, p < .05). (B) Average fold enrichment (H3K4me2/no Ab) from ChIP at *Oct4* in *Kdm1a*^*Vasa*^ (black) and control (white) testes calculated from S4C Fig (n = 3 animals, Unpaired t-test, p < .05). The location of the *Oct4* promoter (prom) primers and the *Oct4* distal (DE) and proximal enhancer (PE) primers are shown below panel A.

KDM1A represses critical genes, such as *Oct4*, during the differentiation of mouse ES cells *in vitro* and hematopoietic stem cells *in vivo* [2, 3]. This raises the possibility that KDM1A could also repress the transcription of *Oct4* during spermatogonial differentiation. Consistent with this possibility, KDM1A binds directly to the *Oct4* locus in mouse ES cells and in a mouse germ cell line (GC-1 cells)[3, 22]. To determine if KDM1A also binds directly to *Oct4* in the mouse testis, we performed KDM1A chromatin immunoprecipitation (ChIP) in wild-type whole adult testes. We observe a 6.3-average fold enrichment of KDM1A at the promoter of *Oct4* compared to a no antibody control (Fig 5A and S4A Fig). There is also significant binding at the proximal enhancer of *Oct4* (a 3.0-average fold enrichment), but no significant binding at the distal enhancer (Fig 5A and S4A Fig). This demonstrates that KDM1A binds directly to *Oct4* in the mouse testis. Importantly since the ChIP analysis was performed on whole testis it is not possible to determine whether KDM1A binding occurs in germ cells or somatic support cells. However, since there are far fewer somatic cells than germ cells in the testis, it is unlikely that the observed enrichment could be explained by KDM1A binding in somatic cells alone [34].

### KDM1A is required for the repression of *Oct4*

**Fig 6:**
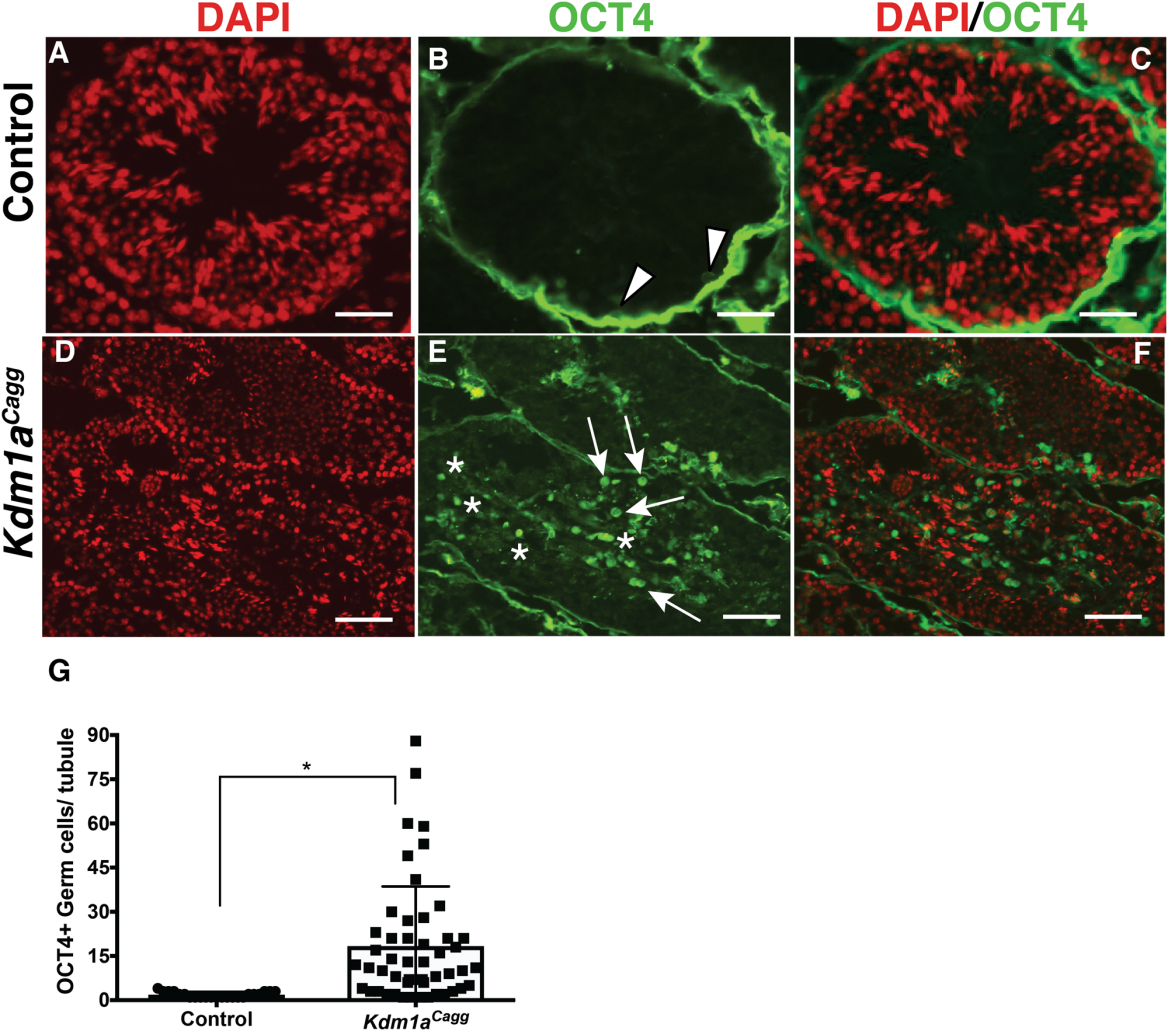
Ectopic expression of spermatogonia genes in *Kdm1a*^*Cagg*^ testes. DAPI (A,D), OCT4 (B,E), and combined (C,F) immunofluorescence from wild-type (A-C) and *Kdm1a*^*Cagg*^ testes (D-F). Arrowheads in (B) indicate spermatogonia marked by OCT4, whereas arrows in (E) indicate OCT4 protein in spermatocyte-like cells. Asterisk (E) indicates OCT4 protein in post-meiotic spermatid-like cells. Quantification of the OCT4 (G) ectopic protein phenotype (n = >50 tubules counted from multiple animals, Mann-Whitney U test, p < .001). Scale bars, 25 μm.

The binding of KDM1A to *Oct4* is consistent with a model where KDM1A is required for the repression of *Oct4* during spermatogenesis. If this is the case, OCT4 might be ectopically expressed in *Kdm1a* mutants. Because *Kdm1a*^*Vasa*^ mutants undergo almost no differentiation, we looked for ectopic expression by performing OCT4 immunofluorescence in *Kdm1a*^*Cagg*^ mutants. In controls, OCT4 protein is confined to spermatogonia (Fig 6A–6C). Likewise, in 28% of the seminiferous tubules from *Kdm1a*^*Cagg*^ mutants, OCT4 is confined to spermatogonia (Fig 6G). However, in the majority of *Kdm1a*^*Cagg*^ mutant seminiferous tubules (72%), OCT4 protein is present throughout the seminiferous tubule in meiotic spermatocyte-like and post-meiotic spermatid-like germ cells (Fig 6D–6F). Quantification of OCT4+ cells demonstrates a 9.1-fold increase in OCT4+ nuclei per seminiferous tubule (*Kdm1a*^*Cagg*^: average 18.4/tubule vs. control: 1.9/tubule)(Fig 6G). This suggests that KDM1A is required to repress *Oct4* expression during spermatogenesis.

### H3K4me2 at the *Oct4* locus

The binding of KDM1A to *Oct4* suggests that KDM1A may repress OCT4 by removing H3K4me2. If this is the case, we would expect an increase in H3K4me2 in *Kdm1a* mutants versus controls. To test this possibility, we performed H3K4me2 chromatin immunoprecipitation (ChIP) on 10-day old whole testes in *Kdm1a*^*Vasa*^ mutants. *Kdm1a*^*Vasa*^ mutants were used, rather than *Kdm1a*^*Cagg*^ mutants, because *Kdm1a*^*Vasa*^ mutants are more uniformly affected. In addition, this analysis was conducted at 10dpp, despite the extensive germ cell loss compared to controls, because at this time point, there is a severe spermatogenesis defect but still a large enough number of germ cells present for ChIP analysis. To account for the fewer germ cells in *Kdm1a*^*Vasa*^ testes, we normalized the fold change of the H3K4me2 ChIP results, based on the difference in percentage of germ cells in *Kdm1a*^*Vasa*^ 10dpp testes versus controls (S4B Fig). After normalization, we detect a large increase in H3K4me2 at the promoter and proximal enhancers of *Oct4* (Fig 5B and S4C Fig). These data suggest that KDM1A may repress OCT4 during spermatogenesis by removing H3K4me2.

## Discussion

Recently, Lambrot *et al*. provided the first evidence that KDM1A functions during the first wave of spermatogenesis [23]. Using an identical combination of conditional *Kdm1a* allele and *Vasa-Cre* transgene, our data agree with their conclusions. Specifically, they find that despite the loss of KDM1A, spermatogonia are still properly established [23]. At 6dpp, we observe no effect on the number OCT4+ spermatogonia. In addition, there are PLZF+ spermatogonia present, though at reduced numbers. These findings confirm the conclusion that KDM1A is not required for the establishment of spermatogonia.

Although KDM1A is not required for the establishment of spermatogonia, Lambrot *et al*. demonstrated that loss of KDM1A has a severe effect on the maintenance and differentiation of spermatogonia [23]. At 6dpp, we show that loss of KDM1A results in a defect in the number of Kit+ and PLZF+ spermatogonia. This defect becomes more pronounced at 8dpp and 10dpp, and by 10dpp we observe many germ cells undergoing apoptosis. We also begin to see a decline in the number of OCT4+ spermatogonia. By 21dpp, loss of KDM1A results in the loss of germ cells. Thus our results confirm the findings of Lambrot *et al*. [23] that KDM1A is required for the maintenance and differentiation of spermatogonia.

To determine if KDM1A also has an ongoing role in adult spermatogenesis, we inducibly deleted *Kdm1a* with the *Cagg-Cre* transgene. Loss of KDM1A during adult spermatogenesis also results in a severe spermatogenesis defect. Specifically we observe a block in the entry to meiosis coupled with a failure to maintain germ cells. These defects in adult spermatogenesis are broadly similar to what we and others [23] observe during the first wave of spermatogenesis, in that in both cases, there is a defect in differentiation coupled to a loss of germ cells. Thus, the function of KDM1A in spermatogenesis appears to be maintained in adult spermatogenesis. Nevertheless, the defect observed during adult spermatogenesis appears to be distinct from the neonatal defect because we also observe the accumulation of meiotic spermatocytes. The accumulation of spermatocytes may be due to activation of a meiotic checkpoint. It is well established that the first wave of spermatogenesis in rodents is distinct from subsequent waves. In particular, during the first wave, prospermatogonia directly become differentiated spermatogonia. It is possible that this difference contributes to the difference in spermatogenesis defects that we observe between neonatal and adult spermatogenesis. In addition, our use of the inducible *Cagg-Cre* transgene makes it possible to detect additional defects due to the distinct timing of deletion in the asynchronous seminiferous tubules. It is possible that this distinct timing enabled us to uncover the meiotic arrest.

The use of the *Cagg-Cre* inducible system enabled us to identify a role for KDM1A in adult spermatogenesis. However, because the *Cagg-Cre* transgene induces KDM1A deletion everywhere, we cannot rule out the possibility that loss of KDM1A in Sertoli cells, or even other tissues, contributes to the observed adult spermatogenesis phenotype. To demonstrate that the *Kdm1a*^*Cagg*^ spermatogenesis phenotype is due to the function of KDM1A in germ cells, we would need an inducible germ cell *Cre* transgene. However, at the moment, no inducible germ cell *Cre* transgene exists. Nevertheless, both we and others have demonstrated a role for KDM1A specifically in germ cells during the first wave of spermatogenesis [23]. Thus, we favor a model where KDM1A is required in germ cells during adult spermatogenesis as well.

Because seminiferous tubules are not synchronized in the adult, the timing of deletion in individual seminiferous tubules enables us to observe potential post-meiotic defects. In both the histology and cleaved caspase staining, we observe round spermatid-like cells that appear to be undergoing apoptosis. In addition, we observe some seminiferous tubules that have round spermatids, but no sperm. These data indicate that some of the *Kdm1a* mutant germ cells that progress through meiosis may still be defective. Thus, there may also be a requirement for KDM1A post-meiotically.

In order to determine why KDM1A is required for spermatogonial differentiation, we investigated two alternative mechanisms. First, based on the similarity of the *Kdm1a* mutant phenotype to *Dnmt3l* and *Miwi2* mutants, we considered the possibility that the observed *Kdm1a* defects are due to meiotic catastrophe caused by the reactivation of retrotransposons. However, we do not detect any defect in the repression of *Line1* or *IAP* retrotransposons. Thus we conclude that the *Kdm1a* phenotype is distinct from *Dnmt3l* and *Miwi2* mutants. This could be because DNMT3L and MIWI2 may act earlier in the germline, before the activation of *Vasa-Cre*. In this case, our experiments could not determine whether KDM1A might also act on retrotransposons earlier. Nevertheless, the similarity between the phenotypic effects in these mutants, hints that they could share a common spermatogenesis checkpoint.

The second potential mechanism that we investigated in our mutants is the failure to repress stem cell gene transcription. If KDM1A is required to repress critical spermatogonia genes during spermatogenesis, then we might expect these genes to be ectopically expressed in *Kdm1a* mutants. In *Kdm1a*^*Cagg*^ mutants, we find that OCT4 protein is maintained in meiotic spermatocyte-like and post-meiotic spermatid-like germ cells. This suggests that KDM1A is required for the repression *Oct4* during spermatogenesis. Consistent with this, we find that KDM1A binds directly to the *Oct4* locus and loss of KDM1A leads to an increase in H3K4me2 at *Oct4*. Based on these data, along with similar expression defects that are observed during differentiation in other stem cell populations [2, 3], it is possible that KDM1A may enable spermatogonial differentiation by repressing the expression of critical genes associated with the undifferentiated cell fate.

The data presented here, as well as in Lambrot *et al*. [23], establish clearly that KDM1A has a critical function during the first wave of spermatogenesis, in the maintenance and differentiation of spermatogonia. In addition, we show here that KDM1A has an ongoing function in adult spermatogenesis, repressing the expression of *Oct4*, enabling meiotic progression and preventing germ cell loss. Taken together, these results demonstrate that KDM1A is an important regulator of spermatogenesis.

## Materials and methods

### Ethics statement

All animal procedures were performed in accordance with the regulations of the NIH Office of Laboratory Animal Welfare and were approved by the Emory University Institutional Animal Care and Use Committee (DAR-2003573-092319N).

### Mice

Mice were housed in a specific pathogen free facility in individually ventilated cages. Mice were given water and diet *ad libitum*. The facility is accredited by the American Association for the Accreditation of Laboratory Animal Care (AAALAC). Mice were monitored daily by members of the laboratory and by animal health technicians. Prior to the experimental endpoint, the mice experienced minimal pain or stress during routine handling and tail biopsies. No animals became ill or died prior to the experimental endpoint. Animals at the experimental endpoint were euthanized by CO_2_ inhalation.

### Generation of KDM1A conditional mutant mice

Generation of the *Kdm1a*^flox/+^ and *Kdm1a*^-/-^ alleles was performed by mating *Kdm1a*^flox/flox^ [14] and *Vasa cre*^+/+^ [25] animals. The resulting *Kdm1a*^flox/+^;*Vasa-cre*^+/-^ males were again mated to *Vasa-cre*^+/+^ females and F2 *Kdm1a*^flox/+^ *Vasa cre*^+/-^ males acquired from this cross were mated to *Kdm1a*^flox/flox^ females to produce *Kdm1a*^-/-^ mutant animals. Primers used for genotyping were: *Kdm1a* floxed (F: 5'-CTCTGTAGCTGTCGAGCTGCTG, R: 5'-GAGGATGGCTCACATTGGTAC), *Kdm1a* deleted (F: 5'-GAACTCCACAGTCATTGATACC, R: 5'-GAGGATGGCTCACATTGGTAC) and Cre (F: 5'-GAACCTGATGGACATGTTCAGG, R: 5'-AGTGCGTTCGAACGCTAGAGCCTGT). Generation of the inducibly deleted alleles was performed by mating *Kdm1a*^flox/flox^ [14] and *Cagg-cre*^+/+^ [26] animals. *Kdm1a*^flox/+^ *Cagg-cre*^+/-^ animals were intercrossed to generate *Kdm1a*^flox/flox^ *Cagg-cre*^+/-^ animals. 10mg/ml tamoxifen (Sigma) in corn oil was administered by intraperitoneal injection at 75mg/kg. Animals were injected once a day on days 1,2,4,5 and 7 (5 times total). Controls were *Kdm1a*^flox/flox^ *Cagg-cre*^-/-^ animals and were injected on the same schedule. In most cases, controls were littermates. In the case that a littermate control was not available an age-matched *Kdm1a*^flox/flox^ *Cagg-cre*^-/-^ control was used.

### Histological methods

For immunofluorescence, testes were fixed for 1–3 hours at 4°C in 4% paraformaldehyde, followed by a 2 hour PBS wash and then transferred to 30% sucrose overnight at 4°C. The tissue was then embedded in O.C.T. compound (Tissue-Tek) and stored at -80°C. 10μm sections were incubated with primary antibody in wash solution (1% heat-inactivated goat serum, 0.5% triton X-100 in PBS) overnight at 4°C in a humidified chamber. Slides were incubated in secondary antibody (1:500 in wash solution) at room temperature for 2 hours in humidified chamber. KDM1A polyclonal Abcam ab17721, SOX9 polyclonal Millipore ab5535, SYCP-1 polyclonal Abcam ab15090, OCT4 monoclonal BD Transduction Laboratories 611202: Fig 5, OCT4 polyclonal Abcam ab19847: Figs 2 and 3, PLZF monoclonal Santa Cruz sc-28319: Fig 5, CLEAVED CASPASE-3 polyclonal Cell Signaling Technology 9661, Alexa Fluor 488 goat-α mouse IgG and, Alexa Fluor 594 goat-α rabibit IgG, Invitrogen.

For histology and immunohistochemistry, testes were fixed in Bouin’s solution overnight, dehydrated in ethanol and xylenes and embedded in paraffin. For histology, 10μm sections were stained with Hematoxylin and Eosin. For immunohistochemistry, antigen retrieval was performed using a microwave in 0.01M Citrate then remaining steps were carried out per manufacturer’s instructions using the VECTASTAIN Elite ABC kit (Vector PK6101). Primary antibodies were diluted in Tris-Cl pH 7.5 with 1% BSA and 0.1% Brij. DAB solution was prepared per manufacturer’s instructions (Vector SK4110) and developed to desired darkness. Slides were then counterstained with hematoxylin (Genetex GTX7334). SOX9 polyclonal Millipore AB5535, PLZF polyclonal Santa Cruz SC22839, KIT polyclonal R&D Systems AF1356. Quantification was performed manually by counting the number of positive cells within seminiferous tubules. Greater than 30 seminiferous tubules per section were analyzed and at least n = 2 animals in all experiments. Statistical analysis was performed using Mann-Whitney U test (p < .05).

### TUNEL assay

TUNEL assays were performed on 10μm frozen sections, per the manufacturer’s specifications (Roche In situ cell death detection kit, Fluorescein).

### In situ hybridization

DIG-labeled in situ hybridization was performed on frozen sections as previously described [35]. Line1 and IAP probes are from Bourc’his et al. [30].

### Bisulfite analysis

Whole testes were digested overnight with Proteinase K in 37° water bath followed by phenol extraction and ethanol precipitation to isolate genomic DNA. Bisulfite conversion was performed with the Zymo EZ DNA Methylation kit, per the manufacturer’s specifications. Bisulfite converted genomic DNA was amplified by PCR and individual TA cloned PCR products were sequenced. Primers: IAP (F: 5’-TTGATAGTTGTGTTTTAAGTGGTAAATAAA, R: 5’-AAAACACCACAAACCAAAATCTTCTAC) and LINE1 (F: 5’ GTTAGAGAATTTGATAGTTTTTGGAATAGG, R: 5’-CCAAAACAAAACCTTTCTCAAACACTATAT). Methylation analysis was performed using BiQ Analyzer software [36].

### Chromatin immunoprecipitation

Whole testes were homogenized in 2-10ml of phosphate-buffered saline with 1% formaldehyde and protease inhibitors (Roche Diagnostics). For adult testes both testes from a single adult animal were used. For neonatal testes, testes were pooled from 3–6 animals. The tissue was homogenized using 10 strokes in a dounce homogenizer and incubated for 10 minutes at room temperature. The cross-linking reaction was terminated by the addition of glycine [final 0.125M]. The samples were centrifuged for 2 minutes at 2000rpm and washed three times with 2-10ml cold PBS with protease inhibitors. After the last wash the pellet was resuspended in 400ul of lysis buffer from the Millipore ChIP assay kit (Millipore) with protease inhibitors. The samples were sonicated for 30minutes (45 second pulse, 15 seconds off on high setting) at 4°C using a Diagenode Bioruptor UCD-200, then centrifuged for 10 minutes at 13,000rpm at 4°C. The supernatant was split equally into two-2ml eppendorf tubes, for immunoprecipitation and for no antibody control. Each sample was diluted to 2ml total volume of ChIP dilution buffer and immunoprecipitation was carried out on 1.5ml, per the manufacturer’s instructions using 10μg of either KDM1A antibody (KDM1A polyclonal Abcam ab17721) or H3K4me2 antibody (H3K4me2 monoclonal Millipore 05–1338). The remaining 500μl was used for an input sample. Precipitated DNA was analyzed using quantitative PCR on a Bio-Rad CFX96 Real-Time PCR machine. Primers: *Oct4* promoter (F: 5'-CTGTAAGGACAGGCCGAGAG, R: 5'-CAGGAGGCCTTCATTTTCAA), *Oct4* proximal enhancer (F: 5'-TCAGGGTAGGCTCTCTGCAC, R: 5'-TCCCCTCACACAAGACTTCC) and *Oct4* distal enhancer (F: 5'-TGAACTGTGGTGGAGAGTGC, R: 5'-GCCAAGTTCACAAAGCTTCC).

## Supporting information

S1 Fig Germ Cell Markers in *Kdm1a*^*Vasa*^ mutants. OCT4+ (A-F), PLZF+ (G-L) and KIT+ (M-R) germ cells from control (A,C,E,G,I,K,M,O,Q) and *Kdm1a*^*Vasa*^ (B,D,F,H,J,L,N,P,R) testes at 6 days post partum (dpp)(A,B,G,H,M,N), 8dpp (C,D,I,J,O,P) and 10dpp (E,F,K,L,Q,R). Images correspond to the quantification in Fig 2. Scale bars, 25 μm.
(JPG)

S2 Fig Germ cells in *Kdm1a*^*Vasa*^ mutants. DAPI (red), and merged (DAPI: red, SOX9: green) from control (A,C,E,G) and *Kdm1a*^*Vasa*^ (B,D,F,H) testes at 6dpp (A,B), 8dpp (C,D), 10dpp (E,F) and 21dpp (G,H) showing germ cells (absence of SOX9). Insets indicate normal spermatocytes (E) and spermatocytes with abnormal morphology (F). Spermatogenic cell types are labeled as described in legend (dpp = days post partum). Scale bars, 25 μm.
(JPG)

S3 Fig Retrotransposon expression and DNA methylation in *Kdm1a* mutants. *IAP* (A,B) and *Line1* (C,D,F,G) in situ (dark purple) hybridization on adult *Kdm1a*^*Cagg*^ (A,C), adult control (B,D), *Kdm1a*^*Vasa*^ 10dpp (F) and control 10dpp (G) testes. Bisulfite analysis at the *Line1* and *IAP* locus in adult *Kdm1a*^*Cagg*^ (E) and *Kdm1a*^*Vasa*^ (H) 10dpp testes versus controls. Circles represent CpG dinucleotides. Filled in circles indicate methylated CpG’s. Hash marks indicate CpG’s not assayed due to sequence alignment. Each row represents an individually TA cloned bisulfite PCR product (E,H). Percentage of CpG methylation at *IAP* and *Line1* in *Kdm1a*^*Cagg*^ (E) and *Kdm1a*^*Vasa*^ (H) testis versus controls is indicated below each diagram. Each methylation analysis was performed on one mutant versus one control. Individual *Line1* and *IAP* clones likely contain different number of CpG residues due to amplification from multiple loci in the genome. Scale bars, 25 μm.
(TIFF)

S4 Fig KDM1A and H3K4me2 chromatin immunoprecipitation. Chromatin immunoprecipitation (ChIP) at *Oct4* (A) showing the percentage input precipitated with a KDM1A antibody (Ab) (black bars) or no Ab (white bars) in wild-type adult testes (n = 2). This data was used to calculate the average fold change in Fig 5A. Quantification of Sertoli cells and germ cells (B) in *Kdm1a*^*Vasa*^ and control testes used for normalization of average fold enrichment in (Fig 5). ChIP at *Oct4* showing the percentage input precipitated with an H3K4me2 Ab (black bars) or no Ab (C) in control versus *Kdm1a*^*Vasa*^ testes at the *Oct4* promoter (prom) and proximal enhancer (PE) (n = 3). This data was used to calculate the average fold change in Fig 5B. Primer locations are the same as the KDM1A ChIP.
(JPG)
