# Supplementary figures and images for: KDM1A/LSD1 regulates the differentiation and maintenance of spermatogonia in mice
Source: PLoS One. 2017 May 12;12(5):e0177473. doi: 10.1371/journal.pone.0177473 (PMC5428937; doi:10.1371/journal.pone.0177473)

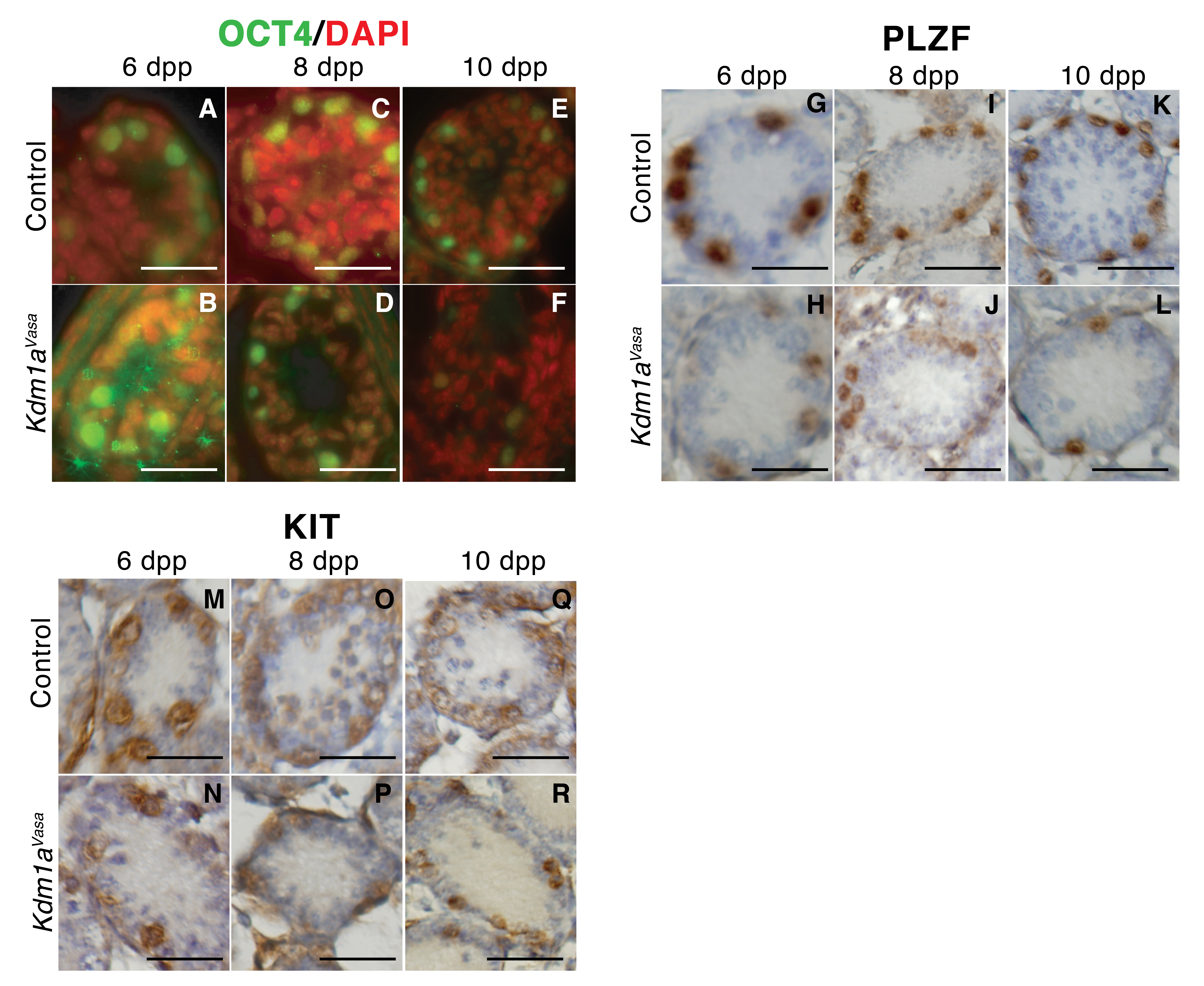

Supplement: S1 Fig — OCT4+ (A-F), PLZF+ (G-L) and KIT+ (M-R) germ cells from control (A,C,E,G,I,K,M,O,Q) and Kdm1aVasa (B,D,F,H,J,L,N,P,R) testes at 6 days post partum (dpp)(A,B,G,H,M,N), 8dpp (C,D,I,J,O,P) and 10dpp (E,F,K,L,Q,R). Images correspond to the quantification in Fig 2. Scale bars, 25 μm. (JPG) [file pone.0177473.s001.jpg]

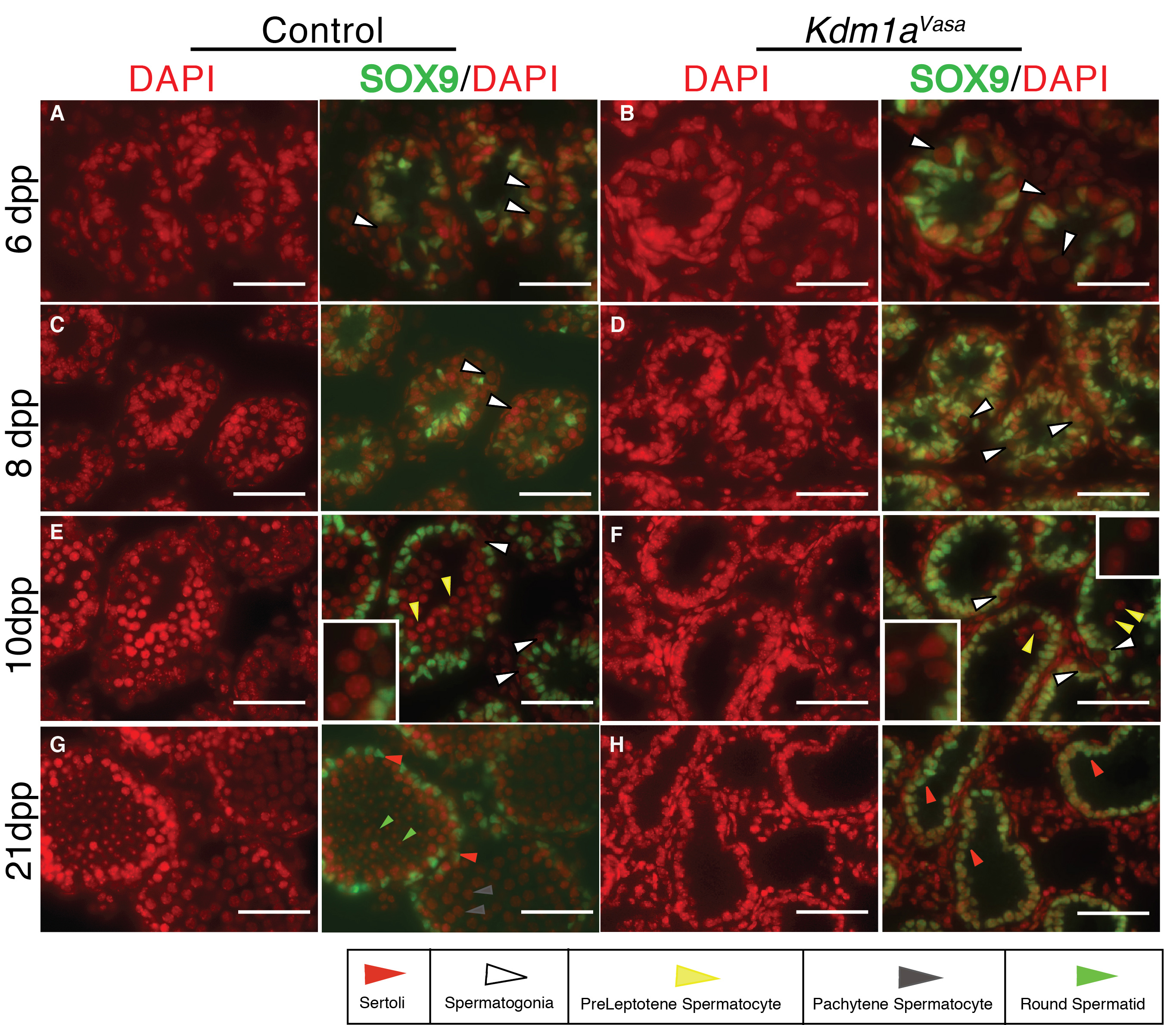

Supplement: S2 Fig — DAPI (red), and merged (DAPI: red, SOX9: green) from control (A,C,E,G) and Kdm1aVasa (B,D,F,H) testes at 6dpp (A,B), 8dpp (C,D), 10dpp (E,F) and 21dpp (G,H) showing germ cells (absence of SOX9). Insets indicate normal spermatocytes (E) and spermatocytes with abnormal morphology (F). Spermatogenic cell types are labeled as described in legend (dpp = days post partum). Scale bars, 25 μm. (JPG) [file pone.0177473.s002.jpg]

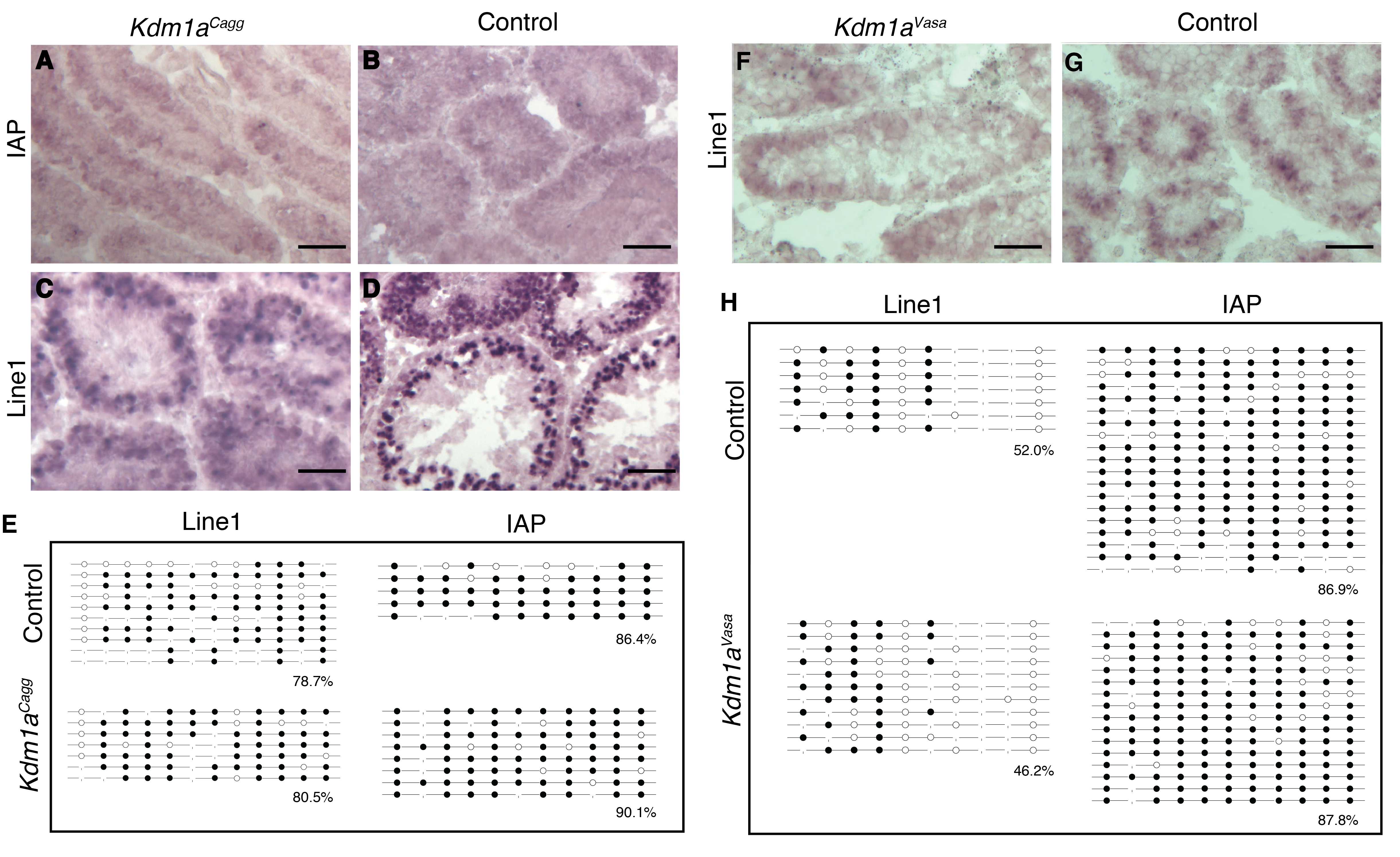

Supplement: S3 Fig — IAP (A,B) and Line1 (C,D,F,G) in situ (dark purple) hybridization on adult Kdm1aCagg (A,C), adult control (B,D), Kdm1aVasa 10dpp (F) and control 10dpp (G) testes. Bisulfite analysis at the Line1 and IAP locus in adult Kdm1aCagg (E) and Kdm1aVasa (H) 10dpp testes versus controls. Circles represent CpG dinucleotides. Filled in circles indicate methylated CpG’s. Hash marks indicate CpG’s not assayed due to sequence alignment. Each row represents an individually TA cloned bisulfite PCR product (E,H). Percentage of CpG methylation at IAP and Line1 in Kdm1aCagg (E) and Kdm1aVasa (H) testis versus controls is indicated below each diagram. Each methylation analysis was performed on one mutant versus one control. Individual Line1 and IAP clones likely contain different number of CpG residues due to amplification from multiple loci in the genome. Scale bars, 25 μm. (TIFF) [file pone.0177473.s003.tiff]

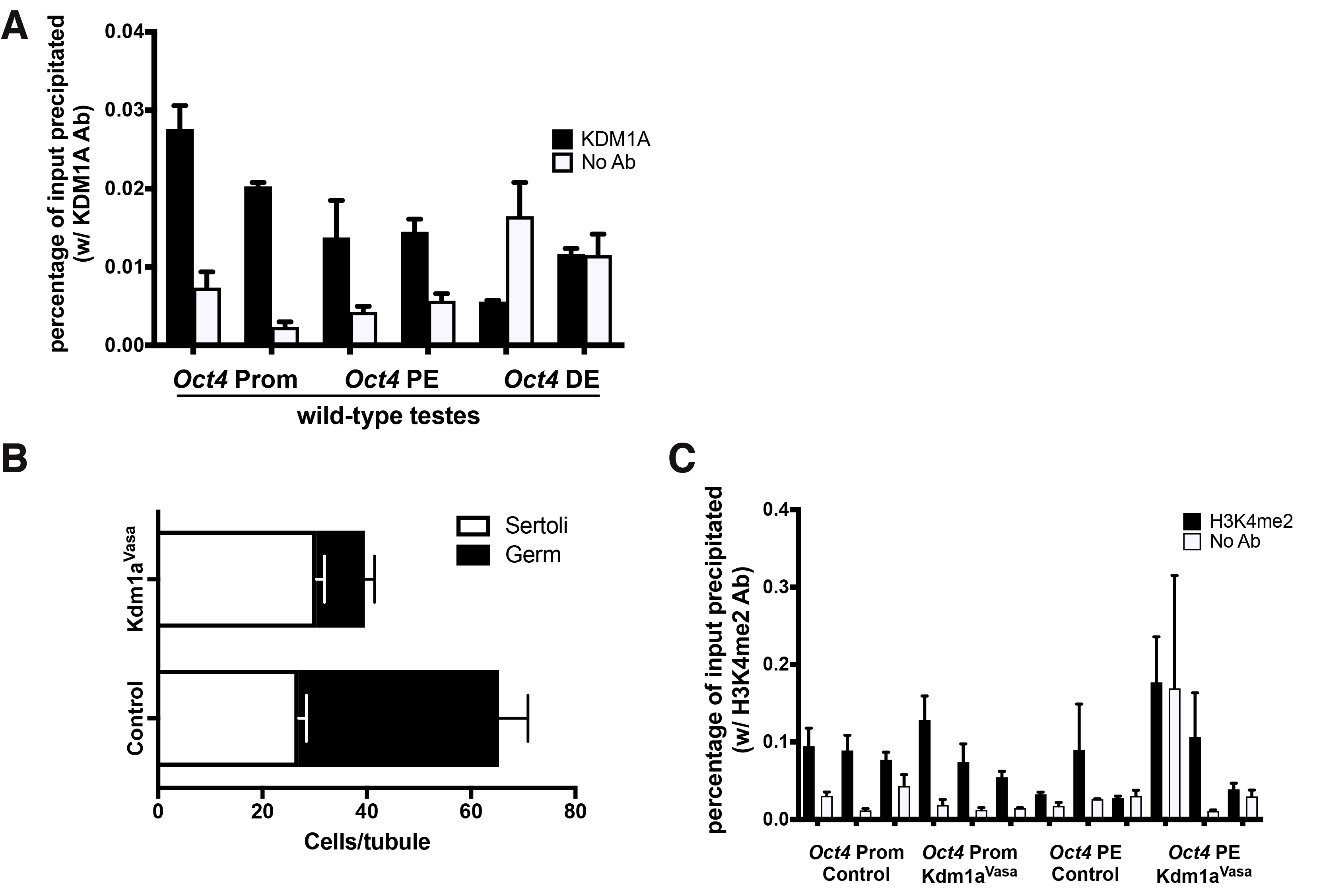

Supplement: S4 Fig — Chromatin immunoprecipitation (ChIP) at Oct4 (A) showing the percentage input precipitated with a KDM1A antibody (Ab) (black bars) or no Ab (white bars) in wild-type adult testes (n = 2). This data was used to calculate the average fold change in Fig 5A. Quantification of Sertoli cells and germ cells (B) in Kdm1aVasa and control testes used for normalization of average fold enrichment in (Fig 5). ChIP at Oct4 showing the percentage input precipitated with an H3K4me2 Ab (black bars) or no Ab (C) in control versus Kdm1aVasa testes at the Oct4 promoter (prom) and proximal enhancer (PE) (n = 3). This data was used to calculate the average fold change in Fig 5B. Primer locations are the same as the KDM1A ChIP. (JPG) [file pone.0177473.s004.jpg]
